# Pathways, prevention, and patient counseling

**DOI:** 10.1007/s12471-026-02040-y

**Published:** 2026-03-11

**Authors:** Pim van der Harst

**Affiliations:** Department of Cardiology, University Medical Centre Groningen, Groningen, The Netherlands

This issue of the *Netherlands Heart Journal* presents three original studies across the spectrum of clinical cardiology involving procedural innovation, long-term secondary prevention, and patient-centred counselling.

In the clinical pathway of pulmonary vein isolation, Scheurwater et al. evaluate replacing manual femoral vein compression with a suture-mediated closure device [1]. Redesigning post-procedural care achieved shorter bed rest, reduced hospitalisation and preserved safety. In an time of increasing atrial fibrillation prevalence and reduced capacity, such refinements can be important logistical adjustments, especially if the estimated cost-savings can be realized.

The second original study focuses on challenges arising after successful percutaneous coronary intervention. Using nationwide data, Timmermans et al. investigated adherence to lipid-lowering therapy [2] and found that restored coronary flow does not guarantee ongoing secondary prevention. Effective long-term risk reduction depends on consistent therapy, but adherence remains poor. Closing the gap between guidelines and actual patient behaviour is crucial for better outcomes.

Hogenhout et al. examined counselling preferences in women with the PLN p.(Arg14del) variant [3]. As preimplantation genetic testing expands to inherited cardiomyopathies, reproductive counselling grows more complex. Participants preferred early, partner-inclusive counselling and were open to testing options. The study shows how cardiology intersects with personal decisions, requiring clinicians to support informed and value-aligned choices.

Together, these 3 contributions illustrate that progress in the field of cardiology is not simply technological or pharmacological. It extends to organisational, behavioural, and relational aspects. Whether refining procedural pathways, strengthening secondary prevention, or guiding advanced genetic counselling, the common denominator is thoughtful, patient-centred care by the professional.
